# A gut feeling about arthritis

**DOI:** 10.7554/eLife.01608

**Published:** 2013-11-05

**Authors:** Diane Mathis

**Affiliations:** 1 **Diane Mathis** is at the Division of Immunology, Department of Microbiology and Immunobiology, Harvard Medical School, Boston, United Statesdm@hms.harvard.edu

**Keywords:** microbiota, inflammation, autoimmunity, rheumatoid, metagenomics, arthritis, Human, Mouse

## Abstract

The gut microbiota of patients recently diagnosed with rheumatoid arthritis is enriched in microbes belonging to the *Prevotella* genus.

**Related research article** Scher JU, Sczesnak A, Longman RS, Segata N, Ubeda C, Bielski C, Rostron T, Cerundolo V, Pamer EG, Abramson SB, Huttenhower C, Littman DR. 2013. Expansion of intestinal *Prevotella copri* correlates with enhanced susceptibility to arthritis. *eLife* **2**:e01202. doi: 10.7554/eLife.01202

**Image** Genomes from the *P. copri* species of gut microbe were abundant in the faeces of patients with rheumatoid arthritis

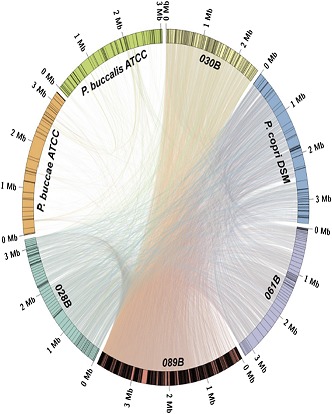

The intestinal microbiota—the complex universe of microbes that are normally resident in the gastrointestinal tract—influences many aspects of life, including glucose metabolism, neural processes, the circadian clock and our immune defenses (Gordon, 2012). It has been known for decades that proper development of the immune system requires the gut microbiota, and that alterations in the repertoire of microbes are often associated with immunological disorders, in particular autoimmune diseases in which the body mistakenly attacks its own tissues (Ivanov and Honda, 2012).

While it is not very surprising that intestinal bacteria affect susceptibility to and/or the severity of autoimmune disorders localized to the gut—notably inflammatory bowel disease—their ability to profoundly impact other immune disorders, including arthritis (Wu et al., 2010), experimental allergic encephalomyelitis (Lee et al., 2011) and type-1 diabetes (Kriegel et al., 2011), came as a surprise. Now, in *eLife*, Dan Littman of New York University School of Medicine and co-workers—including Jose Scher, Andrew Sczesnak and Randy Longman as joint first authors—have taken an important step in extending these findings to humans: they analyzed the faecal microbiota of healthy individuals and of various cohorts of patients with inflammatory arthritis, and documented a striking enrichment of bacteria belonging to the *Prevotella* genus in patients recently diagnosed with rheumatoid arthritis (Scher et al., 2013).

Rheumatoid arthritis affects the body as a whole, but particularly the hands, wrists and other synovial joints (Figure 1) (McInnes and Schett, 2011). It is a multi-factorial disease that has both autoimmune and inflammatory components, and it is subject to diverse genetic, environmental and epigenetic influences. Scher et al.—who are based at various institutions in the US, Italy and the UK—assembled a cohort of subjects who were being treated for chronic rheumatoid arthritis, and a second cohort who had only recently been diagnosed and were therefore not yet receiving treatment. They also recruited subjects being treated for another form of inflammatory arthritis (psoriatic arthritis) and a cohort of healthy controls matched for age and ethnicity.

Scher et al. then cataloged the intestinal microbiota of the four groups by sequencing microbial genes called 16S ribosomal RNA genes—which can be used to distinguish between bacterial species—present in their faeces. They found a marked over-representation of *Prevotella* species, in particular *P. copri*, in most, though not all, members of the recent-onset rheumatoid arthritis group, but in few of the individuals in the other three groups. Conversely, the microbiota of the latter three cohorts was enriched in *Bacteroides* species.

To obtain a more in-depth view, Scher et al. then ‘shotgun’ sequenced the full faecal microbiomes of a subset of the recent-onset rheumatoid arthritis patients and healthy controls. Two important results emerged. First, the gut-associated *P. copri* entities in the controls were not exactly the same as those present in the recently diagnosed rheumatoid arthritis patients. Second, significant differences were observed between the metagenomes (the inventory of genes) of the two cohorts—with the recent-onset rheumatoid arthritis patients showing relatively few genes for enzymes that metabolize vitamins and purines, and relatively many genes for enzymes that metabolize cysteine. While the functional relevance of these differences remains unexplored, they may prove to be useful biomarkers for diagnosis or prognosis.

As a first step in functionally dissecting the association between *P. copri* and autoinflammatory disease, Scher et al. used oral gavage to introduce the bacterium into the stomachs of mice that had been treated with antibiotics. Sequencing of faecal 16S RNA revealed that *P. copri* dominated the gut microbiota within two weeks of introduction. The mice also showed a reduced level of *Bacteroides* (and a few other species), analogous to the findings in the human cohorts. When the *P. copri*-colonized mice were challenged with dextran sulfate sodium, a model for inflammatory bowel disease, they exhibited more severe inflammation of the colon than did control animals.

Scher et al. have taken an important step towards translating to humans recent findings on the gut microbiota–immune system interface obtained from studies on mice. Several lines of investigation now beg pursuit. One critical avenue of study will be to determine whether the association between *P. copri* and rheumatoid arthritis reflects cause, effect or co-association. The fact that the recent-onset rheumatoid arthritis patients alone exhibited high circulating levels of C-reactive protein (a marker of systemic inflammation) raises the possibility that the observed microbiota differences reflect a subclinical inflammatory response, perhaps an immediate precursor of clinical rheumatoid arthritis. More generally, any effects of the disease itself—or of the drugs used to treat the disease—on the microbiota need to be assessed. In humans, certain members of the HLA family of immune-related genes are known to increase the risk of rheumatoid arthritis. The observation that recent-onset rheumatoid arthritis patients with an excess of *P. copri* were less likely to possess some of these risk alleles raises the question of to what extent the association between *P. copri* and rheumatoid arthritis reflects a shared dependence on the HLA locus. Indeed, when human alleles associated with susceptibility or resistance to arthritis were introduced into mice, they had a profound impact on the faecal microbiota, as well as influencing gut permeability and certain populations of intestinal immune cells (Gomez et al., 2012).

Another important avenue of investigation will be to elucidate the mechanisms by which *P. copri* predisposes its host to autoimmunity or inflammation, in particular to a gut-distal disorder. How are arthritogenic signals generated in the gut transposed to the joint? The mysterious microbial world within us is beginning to reveal its secrets, but many mysteries remain to be solved in the years to come.

**Figure 1.**
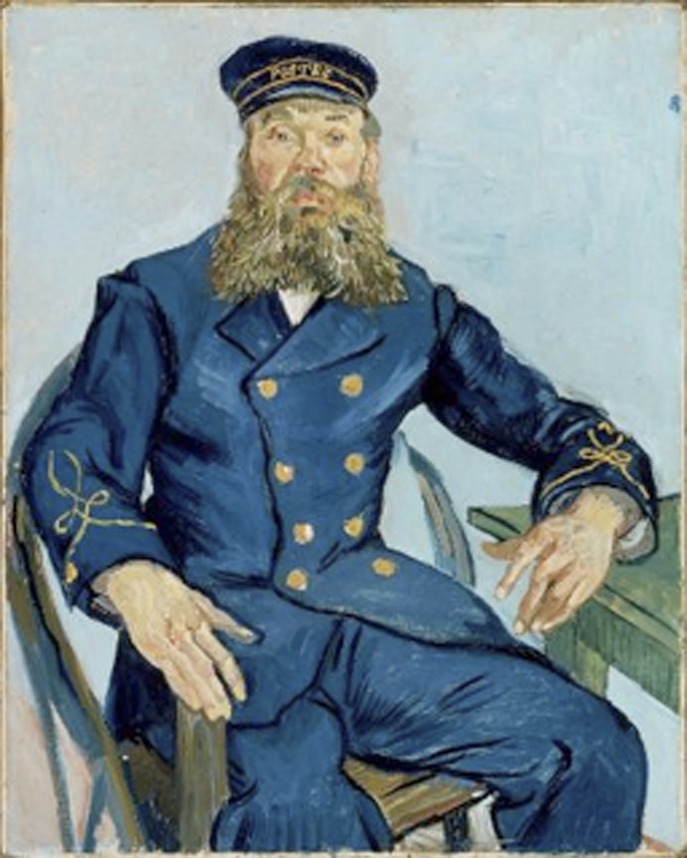
Arthritis as seen by Vincent van Gogh. This painting, “Portrait of the Postman, Joseph Roulin, sitting in a chair” (1888), is one of many portraits that van Gogh painted of individuals suffering from arthritis. The original painting belongs to the Museum of Fine Arts in Boston.

Figure credit: Vincent van Gogh, via Wikimedia Commons
